# Predictors of adherence to screening guidelines for chronic diseases of lifestyle, cancers, and HIV in a health-insured population in South Africa

**DOI:** 10.3402/gha.v7.23807

**Published:** 2014-03-14

**Authors:** Leegale Adonis, Debashis Basu, John Luiz

**Affiliations:** 1 School of Public Health, University of Witwatersrand, Johannesburg, South Africa; 2 Graduate School of Business, University of Cape Town, Cape Town, South Africa

**Keywords:** screening, chronic diseases of lifestyle, cancer, HIV, health insurance, incentives

## Abstract

**Background:**

Adherence to screening guidelines has been widely accepted to reduce morbidity, mortality, and cost outcomes. The aim of this study was to identify predictors of adherence to screening guidelines for chronic diseases of lifestyle (CDL), cancers, and HIV in a health-insured population in South Africa, some of whom voluntarily opt into a wellness program that incentivizes screening.

**Method:**

A cross-sectional study for the period 2007–2011 was conducted using a random sample of 170,471 health insurance members from a single insurer. Adherence to screening guidelines was calculated from medical claims data.

**Results:**

Adherence to screening guidelines ranged from 1.1% for colorectal cancer to 40.9% for cholesterol screening. Members of the wellness program were up to three times more likely to screen for diseases (odds ratio [OR]=3.2 for HIV screening, confidence interval [CI]=2.75–3.73). Plan type (full comprehensive plan) was most strongly associated with cholesterol screening (OR=3.53, CI=3.27–3.80), and most negatively associated (hospital-only core plan) with cervical cancer screening (OR= 0.44, CI=0.28–0.70). Gender was a negative predictor for glucose screening (OR=0.88, CI=0.82–0.96). Provincial residence was most strongly associated with cervical cancer screening (OR=1.89, CI=0.65–5.54).

**Conclusion:**

Adherence to screening recommendations was <50%. Plan type, gender, provincial residence, and belonging to an incentivized wellness program were associated with disproportionate utilization of screening services, even with equal payment access.

Significant disparities exist in preventive healthcare utilization (1). Such disparities are evident not only between high and low-income populations but also within seemingly homogenous populations (2–4). It is well accepted that access to health insurance increases the uptake of screening services (5–7); however, even within the insured population with equal access to services, variation in screening rates exists (8, 9). Inequitable use of healthcare perpetuates the burden of severe illness and mortality in people who tend to need healthcare the most (10). The use of preventive health services, like screening for asymptomatic diseases, effectively reduces morbidity and cause-specific mortality (11–13). Several individual-level factors (level of education, income, age, ethnic minority, or occupation) (14–17) as well as environmental factors (overcrowding, low-income neighborhoods, or lack of a primary care healthcare provider) are negatively associated with the use of screening services (18–20). South Africa is often described as one of the most inequitable countries in the world with marked differences in rates of disease and mortality along racial, gender, provincial, and urban–rural divides as a consequence of its former despotic and poorly functioning government (21–23). Health organizations thus play an important role in improving the overall health status of the population and eliminating health inequality in South Africa.

Discovery Health, the largest health insurance in South Africa with an approximately 40% market share, offers a fully paid screening program for all its members. Some members opt into an incentivized wellness program called Vitality. The Vitality program offers its members incentives to perform various health-screening tests as well as to participate in other health-enhancing behaviors like gym attendance and purchasing healthy foods at partner stores. Screening tests that are incentivized include those for certain cancers (breast, cervix, and prostate), chronic diseases of lifestyle (glucose, cholesterol, BMI, and blood pressure), and the human immunodeficiency virus (HIV). Members of Vitality pay a fee per month (approximately $12.33) and receive benefits, like discounts at network stores, for participating in the program. The Discovery Health screening program is a fully paid for service targeting all of its health-insured members, and the incentives are only offered to those members who join the Vitality program. Screening targets and guidelines used are adapted from the United States Preventative Services Task Force (USPSTF) recommendations.

Even after major health sector reform, South Africa has only some formal population-based screening policies (e.g. cervical cancer screening policy) (24), often poorly implemented with suboptimal uptake (25). A few targeted programs however do operate in selected institutions, provinces, via health insurers and through donor-funded projects. No data exists on the uptake of these programs or the inequities that drive preventive screening behavior in South Africa. This paper aims to identify the factors that impact screening for chronic diseases of lifestyle (CDL), cancers, and HIV in a health-insured population with equal payment access, some of whom are exposed to incentives for screening, specific to South Africa. Knowing the disparities pertaining to preventive care utilization would enable further research agendas, relevant and selective messaging to certain subgroups and could lead to more targeted resource organization and allocation.

## Present investigation

The analysis was a cross-sectional study conducted from 2007 to 2011 based on data collected from Discovery Health member claims using Current Procedural Terminology (CPT) codes. A random 1% sample, not stratified across any variables, consisting of 170,471 members, was generated from the health insurance member database. The study population consisted of all members who were eligible for screening tests (which served as the inclusion criteria), as adapted from the USPSTF recommendations. This is outlined in Table 1. The Vitality program uses these adapted screening recommendations to award points.

**Table 1:** Current USPSTF recommendations and inclusion criteria

Tests	Frequency and age eligibility
Cholesterol	Once every 5 years aged 35+, men aged 20–35 should be screened if they are at increased risk for heart disease
Glucose	Adults once every 5 years; more frequently if BP>135/80
Pap smear	Women aged 21–65 every 3 years. For women aged 30–65 years who want to lengthen screening intervals, screening with a combination of cytology, and human papillomavirus testing every 5 years
Mammogram	Recommendations for 2002: screening mammography with or without clinical breast examination every 1–2 years for women aged 40 and older
Colorectal cancer screening	For adults 50–75 years: Colonoscopy every 10 years and sigmoidoscopy once every 5 years Fecal occult blood: yearly
Prostate specific antigen	Current evidence is insufficient to assess the balance of benefits and harms of screening in men younger than age 75. Screening may be recommended for older men, those with a family history of prostate cancer and African American men who are at increased risk of death from prostate cancer.
HIV	Counseling/screening is recommended for all adults. Frequency of tests not established.
Osteoporosis	Women 65 years and older and in younger women whose fracture risk is equal to or greater than that of a 65-year-old White women who has no additional risk factors. Consideration may be given to women at age 50 years who are post-menopausal and at increased risk.

## Materials and methods

The dependent variables were adherence to the Discovery screening recommendations. These recommendations were classified as follows: 1) receipt of a cholesterol test in the previous 5 years; 2) receipt of a glucose test in the previous 5 years; 3) receipt of a Pap smear in the previous 3 years; 4) receipt of a mammogram in the previous 2 years; 5) receipt of a colon cancer test (fecal occult blood test, sigmoidoscopy, or colonoscopy) in the previous 5 years; 6) receipt of a prostate specific antigen (PSA) test in the previous year; 7) receipt of a HIV test in the previous year; and 8) receipt of a bone scan in the previous year. The following independent variables were studied: sociodemographic factors, such as age and gender; plan type (comprehensive plan: the best benefit package, with higher premiums; core plan: only in-hospital benefits, with the lowest premiums; saver plan (including priority plan): between the comprehensive plan and the core plan in terms of benefits and premiums)^1^; Vitality status (belonging to the wellness program); and province of residence. Current USPSTF recommendations are shown in Table 2. Permission to use the data was provided by the head of research and development at Discovery Vitality and ethical clearance was received from the Witwatersrand Human Research Ethics Committee, certificate number M120854.

**Table 2:** Characteristics of the study sample

Variables	Percentage (%)
Age groups (range of years)	
18–35	38.5
36–50	33.3
51–60	14.5
>60	13.7
Gender	
Male	48
Female	52
Wellness program membership	
Vitality members	64.3
Non-vitality members	35.7
Plan type	
*Core	16.6
**Saver	42.7
***Comprehensive	40.6
Provincial distribution of health insurance membership	
Gauteng	43.4
Western Cape	18.6
KwaZulu-Natal	13.5
Eastern Cape	12.8
Mpumalanga	4.4
North West	2.3
Free State	2.3
Limpopo	1.3
Northern Cape	0.9

* Unlimited private hospital coverage • Essential coverage for chronic medicine • Coverage for medical emergencies when travelling in and outside South Africa. Contributions per month: main member=$153.41; adult dependent=$120.68; child=$61.25.

** Unlimited private hospital coverage • Essential coverage for chronic medicine • A Medical Savings Account • Coverage for medical emergencies when travelling in and outside South Africa. Contributions per month: main member=$206.14; adult dependent=$162.27; child=$82.39.

*** Unlimited private hospital coverage • A choice of a high or no Medical Savings Account and an unlimited Above Threshold Benefit • Comprehensive coverage for chronic medicine • Coverage for medical emergencies when travelling in and outside South Africa. Contributions per month: main member=$350.68; adult dependent=$331.59; child=$70.

Note: KeyCare plan type was excluded from the analyses as the benefit offering differed too much from the other plan types.

### Statistical analysis

A descriptive analysis of all of the study variables was performed. The estimated prevalence for undergoing a screening test during the 2007–2011 study period, with corresponding 95% confidence intervals (CIs) for each screening test, was calculated. An analysis of the association of the study variables with screening adherence was performed using the Chi-squared test statistic and Student's *t*-test, where applicable. Multivariate logistic regression was used to determine which variables were independent predictors of screening for cholesterol, glucose, Pap smears, mammograms, PSA, colorectal cancer, osteoporosis, and HIV. The adjusted odds ratio (OR) and its 95% CI were calculated to measure the strength of the association. A logistic regression multivariate model was built using the forward modeling method. All statistically significant variables (whose bivariate tests were significant) were selected for the multivariate analysis. Calculations were done using the STATA program (Stata Corporation 12.0), and statistical significance was set at *p*<0.05 (*p*-values are two tailed).

## Results

More than 38% of the insured members were aged 18–35 and 33.3% belonged to 36–50 years of age category; both categories had a 52% female distribution. Over 64% of the Discovery Health membership belonged to the Vitality program. The plan types were divided as follows: 40% of the participants were insured through the comprehensive plan, 42% were insured through the saver plan, and 16% were insured through the core plan. The highest proportion of members resided in the better-resourced provinces, as follows: 43% in Gauteng, 18% in Western Cape, and 13% in KwaZulu-Natal. These results are depicted in Table 1.

In this sample data, 40.9% of eligible adults were up-to-date with their cholesterol tests and only 16.6% had taken a glucose test in the preceding 5 years. The cervical screening rate was 11.7% in the previous 3 years, while the breast cancer screening rate was 7.9% in the previous 2 years. The prostate cancer screening rate was 20.6% in the previous year. Just over 2.2% of eligible females had been screened for osteoporosis in the previous year. Only 1.1% of all members aged 50 years and over had been screened for colorectal cancer in the previous 5 years. The HIV screening rate was 8.7% in the previous year. These data, together with eligibility criteria, are shown in Table 3.

**Table 3:** Proportion of members up-to-date with preventive screening 2007–2011

Tests (eligibility criteria for Vitality Screening Program)	Adapted recommendation as per Discovery Health screening program		Proportion of eligible members who screen (%)	Confidence interval
Cholesterol (adults ≥18 years, *n*=20 859)	Adults once every five years		40.9	40.25–41.56
Glucose (adults ≥18 years, *n*=21 517)	Adults once every five years		16.6	16.01–17.07
Pap smear (females ≥16 years, *n*=2529)	Once every three years		11.7	10.5–12.9
Mammogram (females ≥35 years, *n*=827)	Once every two years		7.9	6.1–9.8
Colorectal cancer screening (adults ≥50 years, *n*=4963)	For adults 50 years and older: Colonoscopy and sigmoidoscopy: once every five years Fecal occult blood: yearly		1.1	0.8–1.4
Prostate-specific antigen (males ≥50 years, *n*=107)	Yearly	Mean 2007–2011	27.4	25.6–29.2
		In the previous year	20.6	12.8–28.3
HIV (adults ≥ 18 years, *n*=20 859)	Yearly	Mean 2007–2011	5.4	5.1–5.7
		In the previous year	8.7	8.1–9.3
Osteoporosis (females ≥60 years, *n*=46)	Yearly	Mean 2007–2011	6.6	5.2–7.9
		In the previous year	2.2	1.7–2.7

Health insurance members were 67% more likely to have had a cholesterol test in the preceding 5 years if they belonged to Vitality (OR=1.67), more than 3.5 times more likely if they had a comprehensive plan (OR=3.53), and 42% more likely if they lived in Gauteng province (Gauteng OR=1.42). Comprehensive plan type was also a positive predictor for having had a glucose test in the preceding 5 years, with members being almost twice as likely to have undergone that screening (OR=1.9). The negative predictors of glucose tests were gender (males were 22% less likely to have had a glucose test [OR=0.88]) and core plan type (core plan members were 22% less likely [OR=0.78]).

Females who were members of Vitality were 78% more likely to have had a Pap smear compared to non-Vitality members (OR=1.78); moreover, they were 89% more likely to have had the test if they lived in Northern Cape province (OR=1.89). Female core plan holders were 56% less likely to be up-to-date with their Pap smear tests (OR=0.44). Female Vitality members were 89% more likely to be up-to-date with mammography screening (OR=1.89) and females with a comprehensive plan type were almost three times more likely to be up-to-date with mammography screening (OR=2.99). Core plan type was a negative predictor of mammography screening; females holding that type of plan were 66% less likely to be up-to-date with mammography screening (OR=0.44).

Males over 50 years who were members of Vitality were 45% more likely to have had a prostate cancer screening test (OR=1.45) and were 2.26 times more likely to have had that test if they owned a comprehensive plan (OR=2.26).

The only positive predictor of colorectal cancer (CRC) screening was province of residence, where members were more than 50% more likely to have had CRC screening if they lived in KwaZulu-Natal (OR=1.54) with no negative predictors.

Overall, members of Vitality were more than three times more likely to have had a HIV test (OR=3.2) and they were 27% less likely to have had a HIV test if they owned a core plan (OR=0.73).

Osteoporosis screening decreased by 40% for every 10-year increase in age (OR=0.6).

Table 4 shows all of the predictive variables, their odds ratios, and the corresponding confidence intervals.

**Table 4:** Factors associated with preventive screening

Adjusted OR (CI)									

		Cholesterol	Glucose	Pap smear	Mammogram	PSA	CRC	HIV	Bone scan
Age		Per 10 year increase: 1 (ref)	Per 10 year increase: 1(ref)	Not Significant	Per 10 year increase: 1 (ref)	Per 10 year increase: 1 (ref)	Not significant	Per 10 year increase: 1 (ref)	Per 10 year increase: 1 (ref)
		1.04 (1.03–1.04)	1.3 (1.29–1.35)		1.42 (1.16–1.74)	1.29 (1.16–1.44)		0.73 (0.69–0.76)	0.6 (0.39–0.92)
Gender		Female: 1 (ref)	Female: 1 (ref)	_	_	_	Not significant	Not significant	_
		Male: 1.14 (1.08–1.21)	Male: 0.88 (0.82–0.96)						
Vitality member		Non-member: 1 (ref)	Non-member: 1 (ref)	Non-member: 1 (ref)	Non-member: 1 (ref‘)	Non-member: 1 (ref)	Not significant	Non-member: 1 (ref)	Not significant
		1.67 (1.57–1.76)	1.22 (1.13–1.31)	1.78 (1.4–2.3)	1.89 (1.13–3.17)	1.45 (1.20–1.73)		3.2 (2.75–3.73)	
Plan Type	Core	1.56 (1.44–1.68)	0.78 (0.69–0.86)	0.44 (0.28–0.70)	0.44 (0.17–1.12)	Not Significant	Not Significant	0.73 (0.63–0.98)	Not significant
	Saver	1 (ref)	1 (ref)	1 (ref)	1 (ref)	1 (ref)	1 (ref)	1 (ref)	1 (ref)
	Comprehensive	3.53 (3.27–3.80)	1.9 (1.73–2.12)	Not significant	2.99 (1.72–5.24)	2.26 (1.6–3.1)	Not Significant	0.85 (0.73–0.98)	Not significant
Province		Eastern Cape: 1 (ref))	Eastern Cape 1 (Ref)	Eastern Cape: 1 (ref)		Eastern Cape: 1 (ref)	Eastern Cape: 1 (ref)	Eastern Cape: 1 (ref)	
		Gauteng: 1.42 (1.26–1.61 0.84)	Gauteng: 0.99 (0.82–1.14)	Northern Cape 1.89 (0.65–5.54)	Not Significant	Gauteng 1.22 (0.83–1.79)	KwaZulu-Natal 1.54 (0.49–4.88)	Gauteng 1.32 (0.99–1.77)	Not significant

Adjusted odds ratios using multivariate logistic regression model, adjusted for age, gender, Vitality member, plan type, and Province of residence. OR: odds ratio; CI: confidence interval; Comp: comprehensive plan type; Ref: reference value; PSA: prostate-specific antigen; CRC: colorectal cancer.

## Discussion

Screening rates among this insured population is low. This is not an uncommon finding. Even amongst health insurance members where costs of screening tests are paid for or in settings where screening facilities are easily accessible, researchers have found poor uptake of services (26, 27). Overall, screening rates in this population are far behind comparable international populations. For example, recent 2012 US findings established that 68% of US adults had a cholesterol test in the preceding 5 years (28), and some European countries (Germany, Netherlands, England, and Italy) manage to screen between 61 and 79% of females over 50 years for breast cancer biennially (11).

For this health-insured population, belonging to a wellness program that provides incentives for performing screening tests is a strong predictor of whether a person will undergo cholesterol testing, Pap smears, mammograms, prostate cancer screening, and HIV tests. The use of economic incentives has shown promising results directing patients towards health-enhancing behaviors (29, 30). Positive incentives that reward behavior, rather than negative incentives that are punitive if certain goals are not achieved, tend to achieve greater success (31). Financial incentives can effectively increase the use of preventive services, like mammography screening, Pap smears, and colorectal cancer screening (32), although incentives tend to be more effective for simpler behaviors, like influenza vaccinations, than complex behaviors (29, 33). The type of incentive does not seem as important as the nature of the incentive (i.e. how relevant the incentive is to the person) and researchers are still unsure what size of incentive has the best outcome (33). In this study's population, Vitality members receive discounts at stores for goods like electronic equipment, books, airline flights, car rentals, and discounted holidays. Exposure to incentives in this population may further perpetuate the inequitable use of preventive care services.

The comprehensive plan is one of the most expensive plan types offered by this health insurer, possibly making it affordable for a more affluent part of the membership. This plan type was associated with cholesterol, glucose, Pap smears, mammograms, prostate and cancer. In this population, the finding of a positive association between the comprehensive plan type and some of the screening tests is similar to the findings from other studies that have shown a positive correlation with income level (and level of education), and rates of cervical cancer and prostate cancer screening in particular (34–36). The tests that had the strongest correlations with the comprehensive plan were cholesterol, mammography and prostate cancer screening. In South Africa, breast cancer is more common in the white population (37) and thus this particular population may be requesting/receiving better healthcare screening.

Being male was a negative predictor of glucose screening. This was the only test where males had a lower likelihood of screening whereas in general, males have been shown to use preventive services less often than females (38).

Provincial location was a positive predictor for cholesterol testing, Pap smears, mammograms, PSA, and HIV tests. Geographical variation in the use of screening services occurs in many countries and is often due to unequal resource availability and access as well as differing provider organizations, policies, financial arrangements, skills, and capacity (1, 19, 20, 35). Wide variations exist in healthcare utilization and health outcomes across South African provinces (39). This study further confirms that even in this health-insured community with equal payment access for screening services, provincial location is a strong predictor of service utilization.

Owning a hospital plan (core plan) was negatively associated with glucose screening, Pap smears, mammograms, and HIV tests. Low-socioeconomic status has been implicitly linked to decreased use of preventive health services in many other studies, which can be explained by a combination of factors like low income, lower levels of education, being uninsured, and having limited access to a primary care provider (40). Even in a seemingly homogeneous population (like a health-insured membership such as this), disparities exist in the use of healthcare resources and these are often related to personal predictors (3). The lower socioeconomic strata of this population were shown to be at increased risk of not screening, in particular, for cervical and breast cancer.

Current USPSTF recommendations find no mortality benefit in screening males younger than age 75 and indeed screening may be associated with harms related to over-evaluation (41). Glucose and cholesterol screening has been shown to have no real benefit when implemented at population level, with benefits yielded when screening is targeted at high risk individuals (42, 43). Osteoporosis research has shown that no trials to date have directly evaluated effectiveness, harms, and intervals in females younger than age 65 (44). Although Discovery has adapted some of the USPSTF recommendations to suit their target population, they have to date not undertaken any research to evaluate potential harms that may have occurred as a result of these screening recommendations. Further research is required to fully understand the impact of these recommendations.

## Limitations

This study provides no new information about the predictors of preventive screening services utilization, but highlights vulnerable subgroups of the population specific to the South African health-insured market. The study is a cross-sectional study on the associated factors relating to screening tests and thus cannot make inferences about the direction of the cause-and-effect of the factors found to be associated with those screening tests. The random sample included test for both asymptomatic screening as well as for diagnostic purposes as no distinction could be made based on the CPT code. Screening for asymptomatic diseases may thus be inflated. People self-select into the wellness program creating a selection bias; nevertheless, strong associations were identified for many of the screening tests and belonging to the incentivized program. Screening recommendations are not exactly as per USPTF but an adaptation according to the context of Discovery Health's population and resources. Comparison to other populations’ screening rates thus cannot be done specifically for cardiovascular disease screening. Furthermore, the study's participants represent a very select sample of the South African population and inferences about the results cannot be applied to the general population. However, this is the first study of its kind evaluating predictors of screening behavior in a South African population.

## Conclusion

In this health-insured population with equal payment access for screening services, significant variations exist in the utilization of preventive screening care based on gender, plan type, exposure to incentives, and provincial location. This confirms that payment for screening services alone is not enough to promote utilization. Several other underlying sociodemographic and health resource organizational influences that require further research in this population tend to steer screening behavior.

Owning a certain health plan (either an expensive plan or a cheaper plan) is a predictor of inequitable healthcare utilization, possibly favoring the white affluent member, while males are at particular risk of not screening for chronic diseases of lifestyle.

## Author contributions

LA contributed to the concept, design, and data analysis of the paper. DB and JL both made significant contributions to the interpretation of the results and structure of the research paper. All authors approved the final version for publication.

## Footnotes

1 See Table 1 for description of plan characteristics.
